# Integration of Count Difference and Curve Similarity in Negative Regulatory Element Detection

**DOI:** 10.3389/fgene.2022.818344

**Published:** 2022-02-18

**Authors:** Na He, Wenjing Wang, Chao Fang, Yongjian Tan, Li Li, Chunhui Hou

**Affiliations:** ^1^ Harbin Institute of Technology, Harbin, China; ^2^ Department of Biology, School of Life Sciences, Southern University of Science and Technology, Shenzhen, China; ^3^ School of Life Science and State Key Laboratory of Agrobiotechnology, The Chinese University of Hong Kong, Hong Kong, Hong Kong SAR, China; ^4^ Cancer Centre, Faculty of Health Sciences, University of Macau, Macao, China; ^5^ Department of Bioinformatics, Huazhong Agricultural University, Wuhan, China; ^6^ Hubei Key Laboratory of Agricultural Bioinformatics, Huazhong Agricultural University, Wuhan, China

**Keywords:** count difference, curve similarity, silencer, silencer identification, negative regulatory element

## Abstract

Negative regulatory elements (NREs) down-regulate gene expression by inhibiting the activities of promoters or enhancers. The repressing activity of NREs can be measured globally by massively parallel reporter assays (MPRAs). However, most existing algorithms are designed for the statistical detection of positively enriched signals in MPRA datasets. To identify reduced signals in MPRA experiments, we designed a NRE identification program, fast-NR, by integrating the count and graphic features of sequenced reads to detect NREs using datasets generated by experiments of self-transcribing active regulatory region sequencing (STARR-seq). Fast-NR identified hundreds of silencers in human K562 cells that can be validated by independent methods.

## Introduction

Eukaryotic gene expression is tightly controlled by various types of *cis*-regulatory elements (CREs) that are different in regulatory function, genetic, and epigenetic characteristics (Maston et al., 2006). Promoters and enhancers are positive CREs that initiate and enhance transcription, respectively. Enhancers act locally or over long genomic distances through chromatin looping to regulate their target genes (Shlyueva et al., 2014; Haberle and Stark, 2018; Schoenfelder and Fraser, 2019). In contrast, silencers are negative regulatory elements (NRE) that suppress gene expression through mechanisms that are not completely understood (Ogbourne and Antalis, 1998). Mutations in human CREs associate frequently with tumorigenesis, neurodegeneration, and metabolic diseases (Maston et al., 2006), highlighting the functional importance of transcription control in cells.

In eukaryotic genomes, silencers had not been as vigorously investigated as enhancers (Della Rosa and Spivakov, 2020). Most silencers in the database of silencerDB are predicted (Zeng et al., 2021). Potential silencers were also predicted in cell lines (Doni Jayavelu et al., 2020) by gkmSVM which utilizes sequence features of known silencers (Ghandi et al., 2016). Different from enhancers, silencer prediction is currently infeasible because that whether silencers carry ubiquitous epigenetic signatures is unknown. Genome-wide characterization of functional silencers is thus critical to unveil the genetic and epigenetic features of silencers. Genomic sequences of regulatory activity can be systematically assessed by STARR-seq, a widely used MPRA method initially designed for enhancer identification (Melnikov et al., 2012; Arnold et al., 2013; Crocker and Stern, 2013; Gisselbrecht et al., 2013; Mogno et al., 2013; Vanhille et al., 2015; Wang et al., 2018; Sun et al., 2019). Theoretically, STARR-seq measures silencer activity as well. Actually, Doni Jayavelu et al. had successfully used STARR-seq to measure the transcription-repressing activity of silencers that were predicted by epigenetic features (Doni Jayavelu et al., 2020). Recently, several studies had reported catalogs of silencers that had been predicted or identified by different methods in different model systems at small scales (Petrykowska et al., 2008; Huang et al., 2019; Doni Jayavelu et al., 2020; Pang and Snyder, 2020).

For MPRAs, a statistical method specially designed for silencer identification is needed to facilitate the investigations into silencers’ identity and their roles in transcription regulation. To design a silencer identification program, developers need to consider the functional differences between enhancers and silencers (Zhang et al., 2008; Heinz et al., 2010; Lee et al., 2020). Doni Jayavelu et al. measured silencer activities in selected accessible chromatin regions by comparing the sequenced reads of the reporter cDNA to the reads of the input insert DNA using a one-tail *t* test (Doni Jayavelu et al., 2020). While Pang et al. used a model-based method MAGeCK (Li et al., 2014) after counting reads with the method of HTSeq (Anders et al., 2015; Pang and Snyder, 2020). MAGeCK is similar to edgeR (Robinson et al., 2010) and DESeq (Anders and Huber, 2010) in their design strategies, but it is different from the other two methods in its intended usage. MAGeCK is originally used in CRISPR/Cas9 knockout screen assays. Different from these small-scale assays, genome-wide sequenced reads follow a negative binomial distribution. Potentially, methods designed for the detection of differentially methylated regions (DMRs) or differential chromatin modifications (Shen et al., 2013; Lienhard et al., 2014; Zhang et al., 2014; Lun and Smyth, 2016; Gaspar and Hart, 2017) can be used to identify silencers. However, the specificity, robustness, accuracy, and resolution of these programs have not been evaluated for silencer identification. CRADLE, a recently published method, is designed for enhancer identification (Kim et al., 2021). Theoretically, CRADLE can detect silencers as well. Nevertheless, a computational method designed specifically for the identification of silencers has not been reported.

In this research, we provide a program Fast-NR that is designed for the identification of silencers using STARR-seq-generated datasets by integrating the sequenced read count and signal shape features which are considered in the design of many ChIP-seq peak callers including Polyapeak, PICS, and CLC (Thomas et al., 2017; Hower et al., 2011; Cremona et al., 2019; Yan et al., 2020) (Zhang et al., 2011; Wu and Ji, 2014; Strino and Lappe, 2016). Fast-NR is available at https://github.com/Na-He/Fast-NR. We tested this program on simulated and STARR-seq datasets (Johnson et al., 2018; Doni Jayavelu et al., 2020), compared the performance of Fast-NR with several other programs, and show here that Fast-NR can detect NREs under different conditions.

## Methods

### Algorithm

**FIGURE 1:**
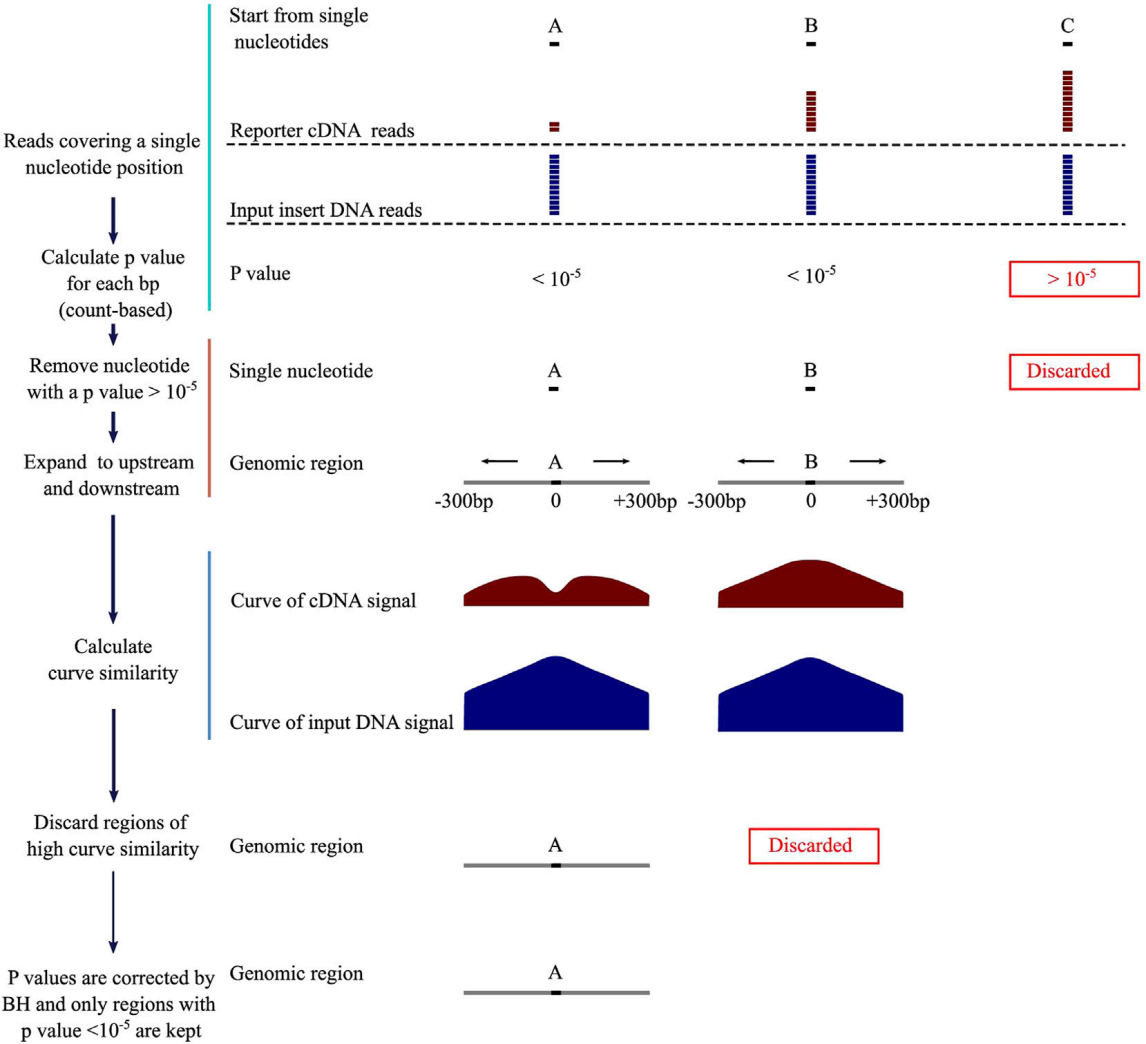
Negative regulatory element identification pipeline. BH, Benjamini–Hochberg correction.

DNA fragments of NRE activity reduce their own expression levels in the STARR-seq reporter cDNA library. To identify NREs, we first calculate *p* values for each nucleotide covered by the reporter cDNA and the input insert DNA across the genome. If a *p* value is below an arbitrary threshold, the corresponding genomic region is considered as a potential NRE. We then plot the numbers of the reporter cDNA and input DNA reads as curves and measure the distances between them to determine whether they are similar by using several different methods. For NREs, the similarity scores are supposed to be low. By integrating count number difference and curve similarity features, we designed a computational method, Fast-NR (Figure 1), and tested its NRE detection power on simulated and real STARR-seq datasets, respectively. Basically, we first screened nucleotides which had the number of reporter cDNA reads smaller than the input insert reads by at least 12, corresponding to the *p* value threshold (10^−5^) we set. We calculate *p* values using cumulative density function (CDF) of negative binomial distribution (NBD). Next, we use the single nucleotides that pass the initial screen as anchors and extend the genomic window to upstream and downstream to a total of 601 bp. We further examine the *p* values of each nucleotide in each 601bp window and keep only windows in which 3/4 of all nucleotides are with a *p* value below 10^−5^. If two windows overlap, we keep the one in the shared region with smaller *p* values. Then, we compare the similarity between the curves of reporter cDNA and the input insert DNA reads, and discard any window with a curve similarity score higher than the arbitrary threshold. Finally, we correct *p* values for each window of identified NRE by Benjamini–Hochberg (BH) test and keep only these with a corrected *p* value smaller than 10^−5^.

### *p* Value Calculation

We calculate *p* values by cumulative density function (CDF) of the negative binomial distribution for the sequenced reporter cDNA reads. The probability mass function of the number of k times failure for a negative binomial distribution is

$$\mathrm{CDF}\left(m,n,p\right)=P\left(x_{n}\leq m\right)=\sum_{i=0}^{m}\left(\frac{i+n-1}{n-1}\right)p^{n}{\left(1-p\right)}^{i},$$

where CDF(m,n,p) returns the probability that is fewer than m times failure before the n th times success, with a single success probability p . Here, the m is treatCount which comes from a negative binomial distribution, n is treatTotal-treatCount, and p is (controlTotal-controlCount)/controlTotal. treatTotal and controlTotal are the total fragment numbers of the reporter cDNA and the input insert DNA in the sequenced libraries, respectively. treatCount and controlCount are the count numbers of reporter cDNA and input insert DNA covering each nucleotide, respectively.

### Curve Similarity

We compare the shape of the curves of the reporter cDNA and the input insert DNA reads. Cosine, Pearson, Euclidean, and an in-house method gradient (linear slope correlation) are used to calculate the curve similarity in this research.

#### Cosine

The method of cosine computes the cosine distance between the 1-D arrays of u and v :

$$\mathrm{Cos}\;\left(u,\;v\right)=1-\frac{\sum_{i=0}^{n}u_{i}v_{i}}{\sqrt{\sum_{i=0}^{n}u_{i}^{2}}\sqrt{\sum_{i=0}^{n}v_{i}^{2}}},$$

where $u_{i}$ and $v_{i}$ are the reporter cDNA and the input insert DNA read count values in the u and v vectors.

#### Euclidean

Euclidean method computes the Euclidean distance between the 1-D arrays of u and v :

$$\mathrm{Euclidean}\left(\mathrm{dis}\right)=\sqrt{\overset{n}{\underset{i=0}{\sum }}{\left(u_{i}-v_{i}\right)}^{2}},$$

where $u_{i}$ and $v_{i}$ are the reporter cDNA and the input insert DNA read count values in the u and v vectors.

#### Pearson

Pearson computes the Pearson correlation coefficient between the 1-D arrays of u and v :

$$\mathrm{cor}=\frac{\sum_{i=0}^{n}\left(u_{i}-\bar{u}\right)\left(v_{i}-\bar{v}\right)}{\sqrt{\sum_{i=0}^{n}{\left(u_{i}-\bar{u}\right)}^{2}}\sqrt{\sum_{i=0}^{n}{\left(v_{i}-\bar{v}\right)}^{2}}},$$

where $u_{i}$ and $v_{i}$ are the reporter cDNA and the input insert DNA read count values in the u and v vectors.

#### Gradient

This algorithm computes the Pearson correlation coefficients between the gradients of the curves of the reporter cDNA and the input insert DNA reads. The coverage and genomic location values of each silencer form a 2-D array, represented by y  and x , respectively. We calculate the gradient between two adjacent points in this array as in the following formula:

$$G\left(i\right)=\;\frac{\left(y_{i+1}-y_{i}\right)}{\left(x_{i+1}-x_{i}\right)},$$

where $y_{i}$ is the coverage value and $x_{i}$ is the position value of the i point in the 2D array. Gradient curve similarity index is the Pearson correlation coefficient (as mentioned above) between the cDNA and the input insert DNA G(i) arrays.

### *p* Value Correction

Bonferroni and Benjamini–Hochberg (BH) adjustments are applied to correct *p* values in our program.

### Datasets

To test the performance of Fast-NR, we downloaded STARR-seq datasets for silencer identification in human K562 (GSE142207) (Doni Jayavelu et al., 2020) and for enhancer identification in untreated A549 cells (GSE114063) (Johnson et al., 2018), respectively. We downloaded histone modification datasets (H3K4me1 GSE91306; H3K27ac GSE91337; H3K4me3 GSE91218; H3K9me3 GSE91335) from ENCODE (Consortium, 2012). H3K27me3 (GSE75903) was downloaded from NCBI (Sayers et al., 2022). We mapped reads to human reference genome version hg19 (GRCh37) using bowtie2 (Langmead and Salzberg, 2012) with default parameters except “-p 24 -X 2000 --sensitive,” then filtered and kept only reads with a value of MAPQ ≥20 by using samtools (Danecek et al., 2021). We kept only unique reads and discarded duplicates by using picardtools (Broad Institute, 2019) except for K562 STARR-seq datasets.

## Results

### Performance of Fast-NR and Other Potential NRE Identification Methods Tested on Simulated Data

We simulated a pair of STARR-seq datasets to test the NRE identification powers of Fast-NR and other methods including csaw (Lun and Smyth, 2016), MEDIPS (Lienhard et al., 2014), PePr (Zhang et al., 2014), and CRADLE (Kim et al., 2021) (see Supplementary Table S1) using default parameters. We used the input insert reads, which are acquired by sequencing plasmids recovered from the transfected cells, and mapped them to chromosome 22 in the enhancer-screening STARR-seq experiment in A549 cells (GSE114063) as the simulation basis. Chromosome 22 is scanned, binned into 400bp windows and only genomic regions covered by at least 100 sequenced reads are kept. We selected 1,000 regions as simulated silencers (true positive silencers) by removing reads from these regions at four different percentage levels (30, 50, 70, and 90%), thus retained fraction of reads at 70, 50, 30, and 10% in the simulated datasets, respectively. These four datasets are used as the reporter cDNA libraries.

**FIGURE 2:**
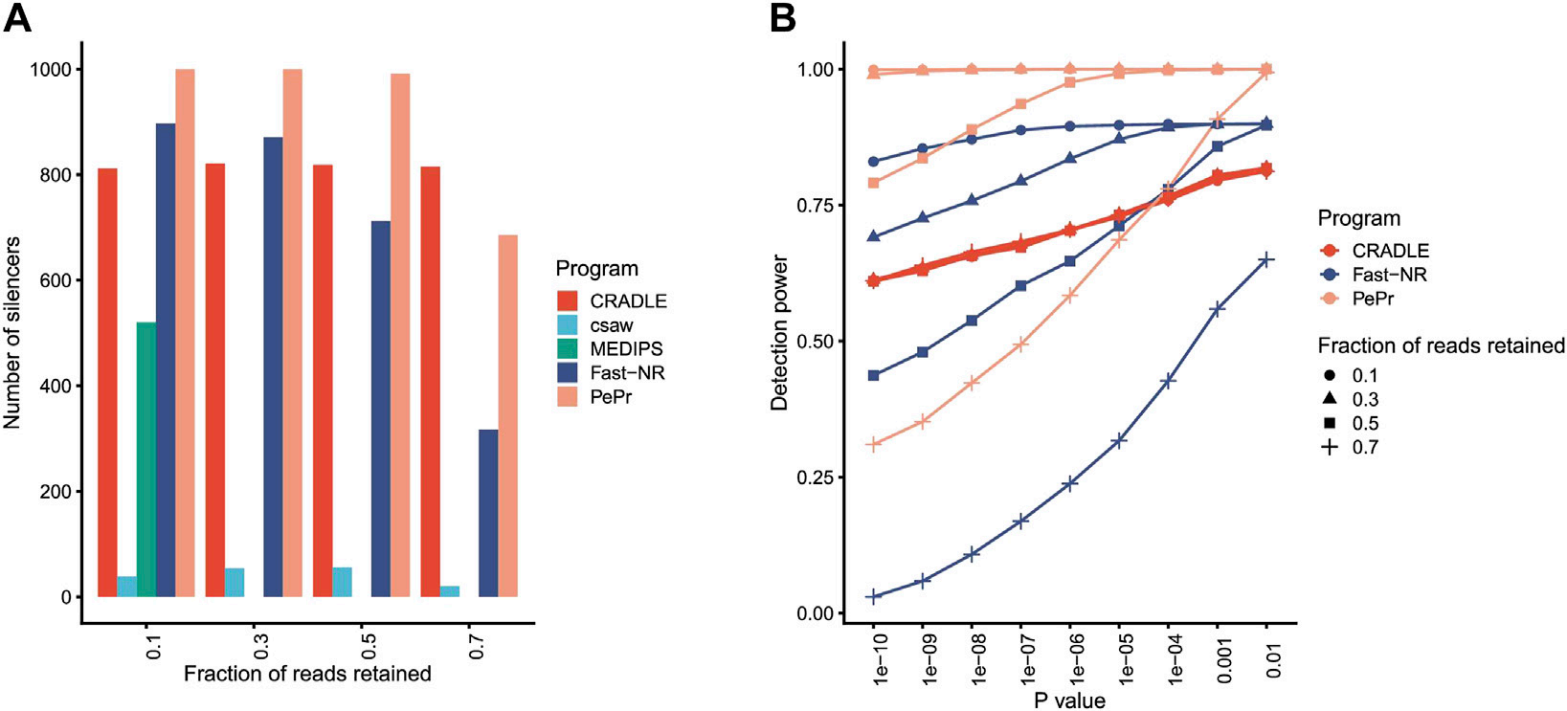
Program performance comparison on simulated datasets. **(A)** The number of silencers identified by different programs. The fraction of reads retained to simulate silencers is shown under *x* axis. *p* value <10^−5^. **(B)** The silencer detection power of different programs at different levels of confidence. Detection power is the ratio between number of identified silencers over total number of simulated silencers.

Program csaw detects differentially enriched regions in ChIP-seq dataset by using a sliding window strategy. MEDIPS identifies differentially methylated sites in the dataset generated by methylated DNA immunoprecipitation sequencing (MeDIP-seq). MEDIPS fails to detect any silencer at all from libraries in which 30, 50, and 70% reads are retained for the simulated silencers, while csaw identifies less than 100 silencers independent of what percentages of reads are retained (Figure 2A). These results suggest that MEDIPS and csaw may lack NRE detection power.

Program PePr is similar to csaw in their differential signal detection power for ChIP-seq datasets. PePr identifies most simulated silencers when reads are retained at three different levels (10, 30, and 50%) (Figure 2A), suggesting PePr could potentially be a usable NRE identification method. Program CRADLE calls both positive and negative regulatory elements for STARR-seq datasets. This program identifies approximately 800 silencers (815, 819, 821, and 812, respectively) independent of the percentages of reads retained (Figure 2A). Fast-NR detects 897, 871, 712, and 317 simulated silencers at 10, 30, 50, and 70% retained read levels (Figure 2A), suggesting its NRE identification power correlates positively with the read removal percentages. These results together suggest that PePr, CRADLE, and Fast-NR may all be usable NRE identification methods. Also, Fast-NR is more sensitive to signal reduction levels than other programs.

The NRE detection power of PePr, CRADLE, and Fast-NR may change when different *p* value thresholds are applied. Indeed, all these three methods detect fewer silencers as the *p* value threshold becomes more stringent (Figure 2B). Again, CRADLE is insensitive to the read removal percentage. Interestingly, though Fast-NR detects fewer silencers as *p* value decreases, it identifies more silencers than CRADLE when 10 and 30% of reads are retained. PePr is also sensitive to the change of *p* value threshold, especially when the fraction of reads retained is 70% (Figure 2B). These results show PePr and Fast-NR are more sensitive to the read retained rates than CRADLE. However, these results do not suggest which program outperforms the others.

### Performance of Fast-NR and Other Potential NRE Identification Methods Tested on Real STARR-Seq Datasets

**FIGURE 3:**
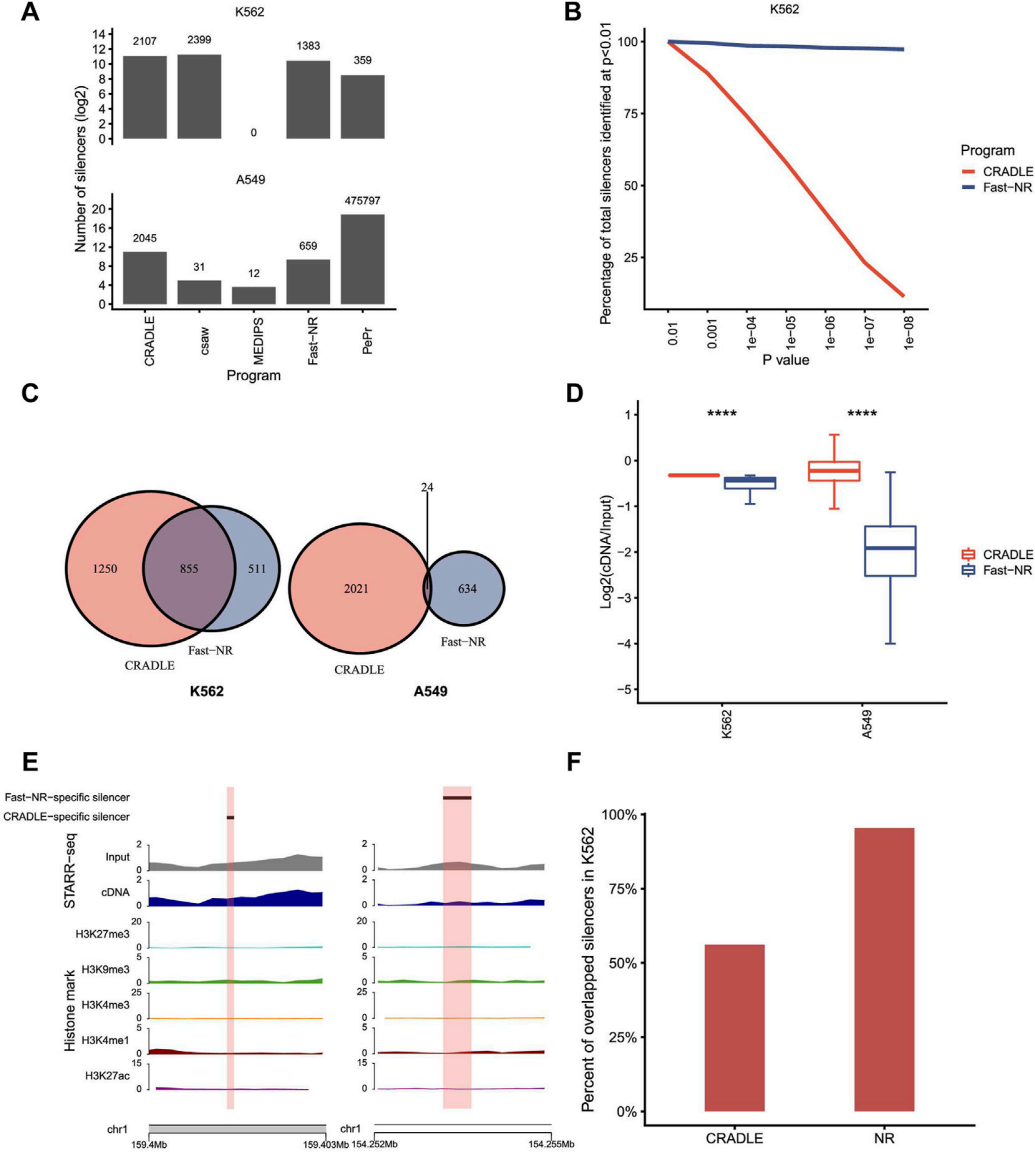
Program performance comparison on STARR-seq datasets. **(A)** The number of silencers identified by different programs. *p* value <10^−5^. **(B)** The percentage of silencers identified by CRADLE and Fast-NR at different confidence levels. The number of silencers identified at *p* < 10^−2^ is set at 100%. **(C)** Venn diagrams of silencers identified by Fast-NR and CRADLE in K562 and A549, respectively. *p* value <10^−5^. **(D)** Reads ratio (reporter cDNA/input inserts) distribution for silencers identified only by CRADLE or Fast-NR in K562 and A549, respectively. **(E)** Exemplary silencers identified only by CRADLE (left) or by Fast-NR (right). **(F)** Percentages of Fast-NR- and CRADLE-identified silencers (*p* < 10^−5^) reported by Doni Jayavelu et al.

Theoretically, a genomic region of repressing activity is supposed to be transcribed less and underrepresented in the reporter cDNA library of STARR-seq. We downloaded STARR-seq datasets for silencer and enhancer identifications in human K562 (Doni Jayavelu et al., 2020) and A549 (Johnson et al., 2018) cells, respectively (Supplementary Table S2). STARR-seq in K562 measures the repressing activity of 7,430 sites in the accessible regions that are predicted as potential silencers based on epigenetic states. Differently, STARR-seq in A549 cells measures enhancer activities genome wide. We tested the NRE detection power of the five programs on the datasets generated by these two STARR-seq experiments (Figure 3A). Both Fast-NR and CRADLE identified hundreds and thousands NREs. Program csaw identified 2,399 silencers in K562 and only 31 silencers in A549. PePr identified 359 NREs in K562, but unbelievably, 475,797 NREs in A549. MEDIPS nearly failed to identify any NREs in the two STARR-seq experiments. These results confirm that MEDIPS lacks the NRE detection power for either simulated or real experimental data. Programs csaw and PePr perform differently on the two STARR-seq experiments, and their poor consistency in performance compromised our confidence to use them for NRE identification. After these comparisons, we kept Fast-NR and CRADLE for more evaluation analyses.

We compared Fast-NR and CRADLE’s performance by changing the *p* value threshold for NRE identification. For K562 STARR-seq, Fast-NR identified silencers consistently at high levels and was nearly not affected by the change in the *p* value threshold (Figure 3B). In contrast, the number of silencers identified by CRADLE dropped dramatically to only about 10% (*p* < 1 × 10^−8^) of these identified at *p* < 0.01. These results provoked us to examine the overlapping rates of silencers identified by Fast-NR and CRADLE in K562. In fact, decent amounts of silencers identified by these two methods overlapped in K562 but not in A549 (Figure 3C). Silencers identified were expected to have lower cDNA reads than the input insert DNA reads. We calculated the ratios of (cDNA reads)/(insert reads) for CRADLE-specific and Fast-NR-specific silencers in K562 and A549. The CRADLE-specific silencers showed less reduction in cDNA reads than Fast-NR-specific silencers (Figures 3D,E). Over 95% (1,320/1,383) of Fast-NR identified silencers in K562 overlapped with the reported silencers (Doni Jayavelu et al., 2020) (Figure 3F). In contrast, only 56% silencers identified by CRADLE overlapped with the reported silencers (Figure 3F). These results suggest that many CRADLE-specific silencers were identified because of the heavy correction procedures that are integral to CRADLE (Kim et al., 2021). These CRADLE-specific silencers seemed to be “false-positives” in terms of the reduction rate in the reporter cDNA reads.

To reveal which transcription factors may bind to silencers, we searched through the sequences of silencers and identified a few DNA motifs enriched for transcription factors binding (Supplementary Table S3). One of these motifs was the silencing factor REST binding site (Chong et al., 1995), which was particularly enriched in Fast-NR identified silencers. DNA motif for transcription repressor PRDM6 was also enriched (Davis et al., 2006). Histone H4K20 methylation is a mark reported to be associated with silencers (Pang and Snyder, 2020). The binding motif (GC-box sequence) for the transcription factor of Sp1-like factors was also enriched in both Fast-NR and CRADLE silencers. Sp1-like factors activate or repress transcription in response to different physiological and pathological stimuli (Zhao and Meng, 2005). DNA motifs of PAX5 and FOS were enriched at Faste-NR and CATDLE silencers as well. Many transcription factors have dual functional roles in gene regulation, and silencers have been reported to be switchable to enhancers during development and in different cell types (Bessis et al., 1997; Cavalli, 2014; Gisselbrecht et al., 2020). Enrichment of any specific transcription factor’s binding motif may not necessarily correlate with the regulatory activity of a CRE in a specific cell type. Nevertheless, silencers are indeed enriched with certain DNA motifs for transcription repressors in our analysis, suggesting that silencers identified by Fast-NR are very likely to be biologically functional.

### Curve Similarity Analyses in Fast-NR

**FIGURE 4:**
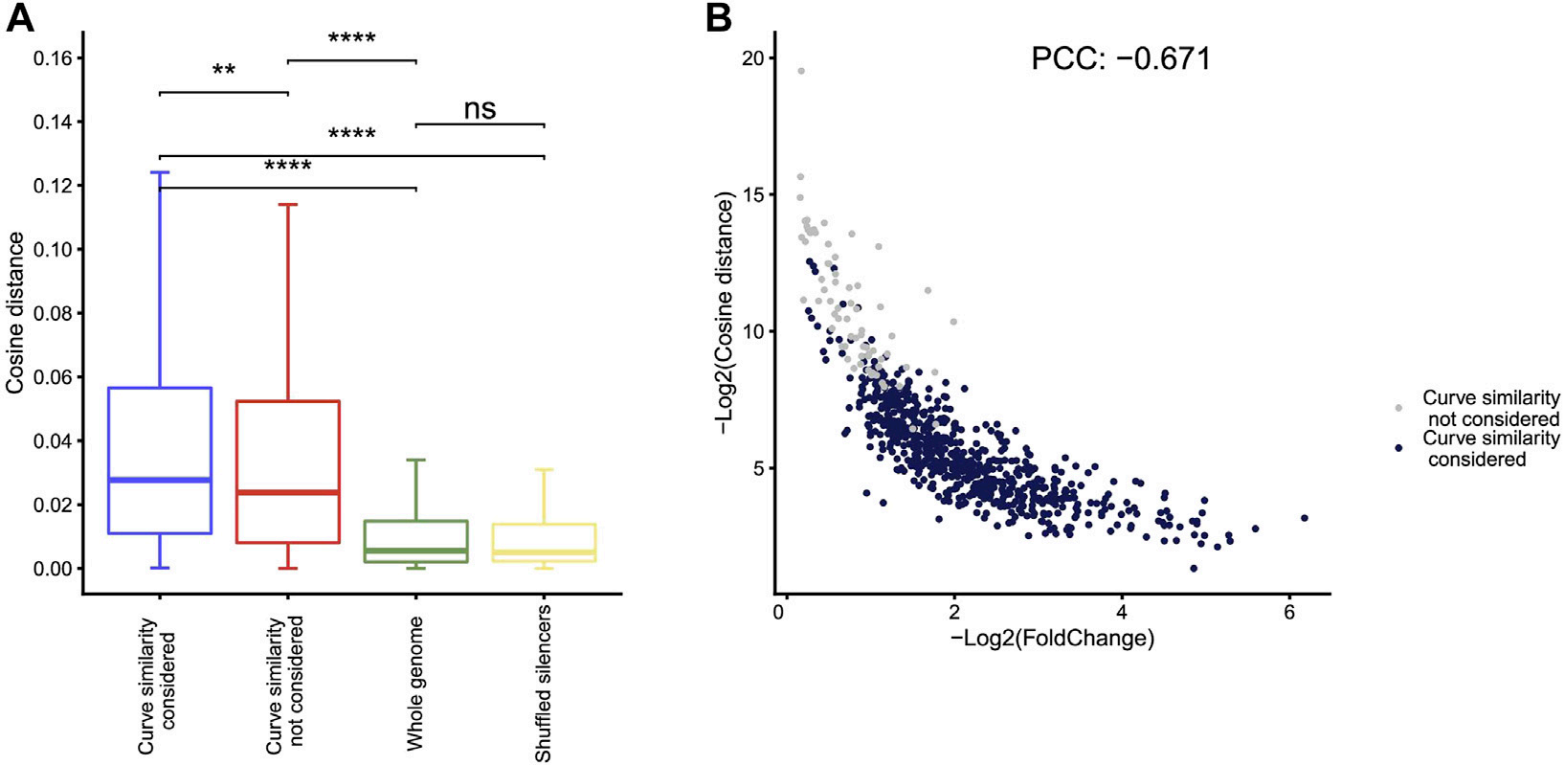
Curve similarity effect on silencer identification. **(A)** The cosine distance distribution for silencers identified by Fast-NR with similarity considered, similarity not considered, and controls of whole genome regions with 400 bp size and shuffled silencer regions. Distance negatively correlates with similarity. **p < 10^−3^, ***p < 10^−4^, Wilcoxon rank sum test. **(B)** The correlation between silencer strength and curve similarity. The *X* axis shows the value of −log_2_ (cDNA reads/insert DNA reads). The *Y* axis shows the curve similarity index, −log_2_ (Cosine distance) of silencers calculated by the method of cosine. Blue dots are silencers that pass curve similarity threshold of 0.9, and gray dots are silencers that do not pass curve similarity threshold.

Compared to the other four methods, Fast-NR is the only one that takes into account the similarity between the curves of the reporter cDNA and the input insert DNA signals. We examined to what extent the similarity between the curves of the reporter cDNA and the insert DNA reads could affect the NRE identification. We calculated the similarity index values (−log_2_CosineDistance) for the NREs identified in A549 and found that the cosine distances between cDNA and plasmid curves are much higher than the random chosen genomic control regions (Figure 4A). Interestingly, the similarity index values correlate negatively with the strengths of silencers (Figure 4B), suggesting stronger silencers have low curve similarity. We obtained similar results using other curve similarity calculation methods such as Pearson, Euclidean, and gradient (Supplementary Figure S1A).

We removed the curve similarity requirement in Fast-NR and identified more silencers (gray dots in Figure 4B). These “new” silencers have high curve similarity index values and low silencer strengths compared to the silencers identified with curve similarity considered (blue dots in Figure 4B). Interestingly, curve similarity correlated poorly with *p* values (Supplementary Figure S1B), suggesting curve similarity in Fast-NR is a feature independent from the ratio between the reporter cDNA and the input insert DNA reads. The Pearson’s correlation coefficients between the curve similarities and the silencer activities could be positive or negative depending on the method used (Supplementary Figure S1C). These results together show that curve similarity comparison is also important for the reliable identification of NREs.

## Discussion

In this study, we presented a program of Fast-NR, in which both read counts and shape similarity are considered, for the detection of NREs using STARR-seq datasets and compare its performance with other four programs of csaw (Lun and Smyth, 2016), MEDIPS (Lienhard et al., 2014), PePr (Zhang et al., 2014), and CRADLE (Kim et al., 2021). Among them, MEDIPS, designed for DNA methylation analysis, shows worst compatibility with silencer identification on either simulated or experimentally generated datasets. Programs csaw and PePr detect significantly differential regions in ChIP-seq data. Neither of them performs consistently when being applied to different types of datasets. Besides methods tested in this research, other methods designed for the identification of differentially enriched signals are not suitable for silencer identification either. For example, DMRfinder (Gaspar and Hart, 2017), DSS (Park and Wu, 2016), and HMST-Seq-Analyzer (Farooq et al., 2020) require specific input data format that are not compatible with NRE analysis.

Fast-NR and CRADLE seem to be good choices for both simulated and experimentally generated datasets. However, many silencers identified by CRADLE showed only small reduction in the reporter cDNA signal than the input insert DNA, and curves of these signals were highly similar. CRADLE uses the GLM approach to correct four types of bias, the DNA structure affecting shear force, Gibbs free-energy affecting PCR efficiency, read sequences mappability, and G-quadruplex affecting DNA polymerase processivity (Kim et al., 2021). CRADLE treats the corrected signals as normal distribution and uses Welch’s *t*-test to search for differences. As shown in our analysis, these corrections lead to the detection of “silencers” that cannot be identified based on the differences in the read counts of the reporter cDNA and the input insert DNA.

Being different from methods using sliding window strategy, Fast-NR detects the difference in the number of reporter cDNA and input insert DNA reads at single base-resolution. It is potentially possible to use Fast-NR to reveal the precise locations of regulatory elements and the binding sites of transcription factors.

STARR-seq tests silencers activity in episomal reporter plasmids independent from the endogenous chromatin environment. Ideally, regulatory activities of potential CREs can be tested in their proper chromatin context. Methods for endogenous CREs analysis, such as multiplexed editing regulatory assay (MERA) (Rajagopal et al., 2016) and thousands of reporters integrated in parallel (TRIP) (Akhtar et al., 2013), can be used to measure the regulatory activities of genomic regions in the native cellular context. However, these methods are generally not applicable for unbiased genome-wide analysis of CREs. Nevertheless, the combination of these methods and STARR-seq will help to achieve a global identification, and at the same time, a large scale endogenous validation of CREs.

Another issue we would like to point out is the promoter used in the reporter plasmids. In STARR-seq and related methods, promoter choice could affect the outcome because the promoter used may be irresponsive to some CREs. We speculate that using promoters of house-keeping genes and cell type-specific genes may allow the identification of more CREs that may prefer to regulate different types of promoters. To save the computation time, we filtered potential NREs by applying thresholds on both read counts and *p* values sequentially, which may also, theoretically, reduce false positive rate. However, the thresholds applied could be too strict and exclude some true silencers. We recommend the users to test the threshold effects and choose appropriate thresholds for their own analysis.

Though STARR-seq measures regulatory activity of tested DNA fragment in episomal environment, it provides a catalogue of CREs that can be further tested at their endogenous loci by alternative methods. We did not take sequencing bias into consideration because experimental data that can be used to determine to what extent biases may affect NRE identification were not available. In summary, by combining read count-based negative binomial test and shape similarity comparison, we have shown that Fast-NR is potentially usable for silencer identification, thus providing a powerful and robust computational method for NRE identification.

## Conclusion

Silencers are negative regulatory elements that control the precise gene expression during cell proliferation and differentiation. The increasing needs for global silencer characterization require a reliable and user-friendly computational method. Our method Fast-NR integrates single nucleotide read count information and graphic information to detect silencers genome widely. Fast-NR identifies NREs at single base resolution. The wide application of Fast-NR will accelerate the genetic and epigenetic studies of the intriguing functional mechanisms of silencers.

## Data Availability

The original contributions presented in the study are included in the article/Supplementary Material; further inquiries can be directed to the corresponding authors.
