# Supplementary figures and images for: Integration of Count Difference and Curve Similarity in Negative Regulatory Element Detection
Source: Front Genet. 2022 Feb 18;13:818344. doi: 10.3389/fgene.2022.818344 (PMC8896116; doi:10.3389/fgene.2022.818344)

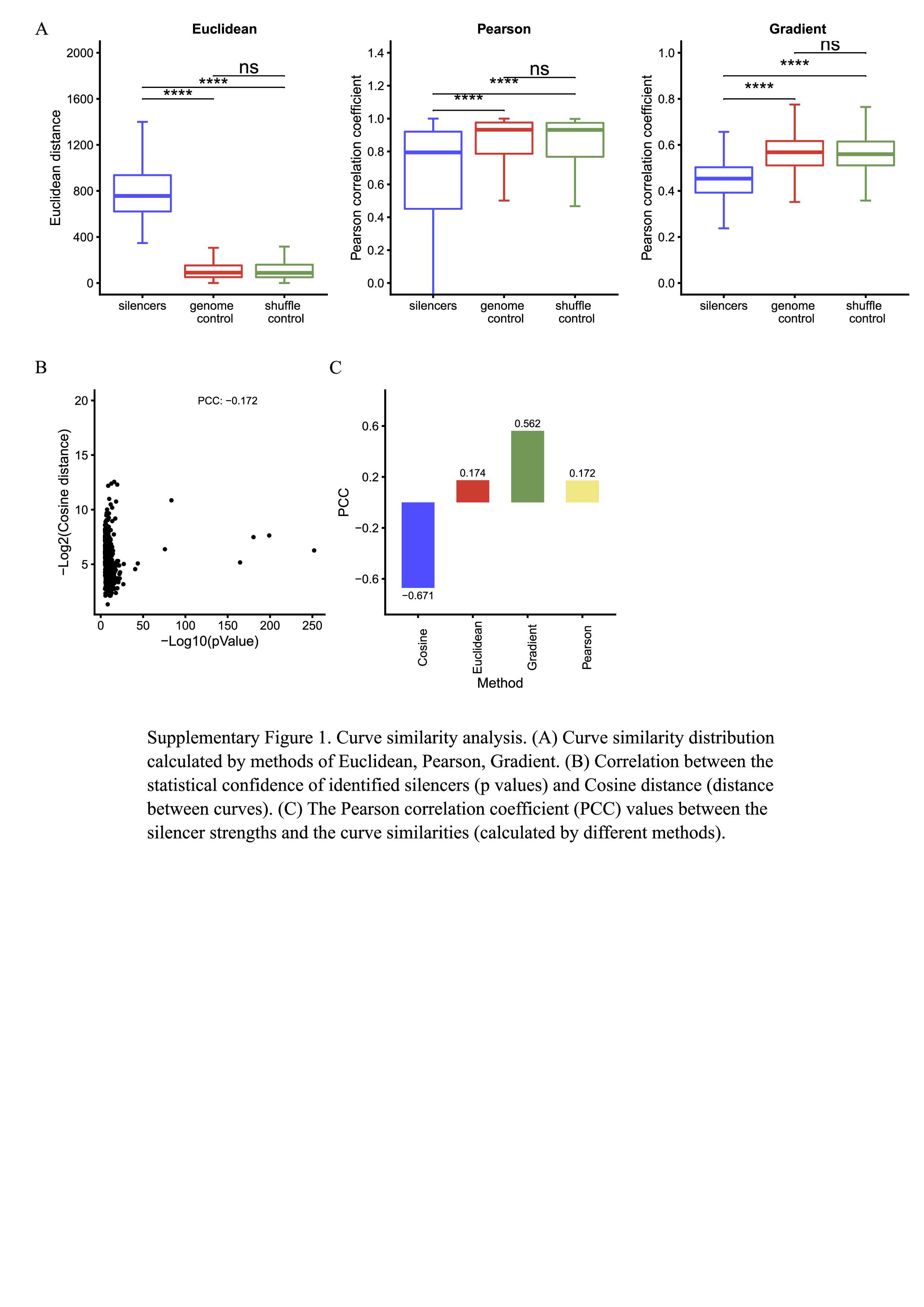

Supplement: Supplementary file 3 [file Image1.JPEG]
